# The American Popular

**Published:** 1875-11

**Authors:** 


### THE AMERICAN POPULAR.

Last month we had a few words to say on the subject of Life Insurance, with reference to the method of classification adopted by the AMERICAN POPULAR LIFE INSURANCE COMPANY, of this city. We then asserted our belief in the system which selects the very best risks to insure at low rates, and places the bad ones also by themselves, at increased charges. Of course this commendation of the course pursued by this company attracted their attention, and, while not complaining of our method of treating the subject, they seem to prefer to speak for themselves. This they do in an advertisement of three pages at the close of the present number. To the company's presentation of the case we invite the attention of thoughtful readers.

